# Proteomic profiling of soft tissue sarcomas with SWATH mass spectrometry

**DOI:** 10.1016/j.jprot.2021.104236

**Published:** 2021-06-15

**Authors:** Martina Milighetti, Lukas Krasny, Alex T.J. Lee, Gabriele Morani, Cornelia Szecsei, Yingtong Chen, Nafia Guljar, Frank McCarthy, Christopher P. Wilding, Amani Arthur, Cyril Fisher, Ian Judson, Khin Thway, Maggie C.U. Cheang, Robin L. Jones, Paul H. Huang

**Affiliations:** a Division of Molecular Pathology, The Institute of Cancer Research, 237 Fulham Road, SW3 6JB London, UK; b Sarcoma Unit, The Royal Marsden NHS Foundation Trust, 203 Fulham Road, SW3 6JJ London, UK; c Division of Clinical Studies, The Institute of Cancer Research, 15 Cotswold Road, SM2 5NG Sutton, London, UK; d Clinical Trials and Statistics Unit, The Institute of Cancer Research, 15 Cotswold Road, SM2 5NG Sutton, London, UK

**Keywords:** Soft tissue sarcoma, Proteomics, Mass spectrometry, SWATH MS, FFPE, Biomarkers

## Abstract

Soft tissue sarcomas (STS) are a group of rare and heterogeneous cancers. While large-scale genomic and epigenomic profiling of STS have been undertaken, proteomic analysis has thus far been limited. Here we utilise sequential window acquisition of all theoretical fragment ion spectra mass spectrometry (SWATH-MS) for proteomic profiling of formalin fixed paraffin embedded (FFPE) specimens from a cohort of STS patients (*n* = 36) across four histological subtypes (leiomyosarcoma, synovial sarcoma, undifferentiated pleomorphic sarcoma and dedifferentiated liposarcoma). We quantified 2951 proteins across all cases and show that there is a significant enrichment of gene sets associated with smooth muscle contraction in leiomyosarcoma, RNA splicing regulation in synovial sarcoma and leukocyte activation in undifferentiated pleomorphic sarcoma. We further identified a subgroup of STS cases that have a distinct expression profile in a panel of proteins, with worse survival outcomes when compared to the rest of the cohort. Our study highlights the value of comprehensive proteomic characterisation as a means to identify histotype-specific STS profiles that describe key biological pathways of clinical and therapeutic relevance; as well as for discovering new prognostic biomarkers in this group of rare and difficult-to-treat diseases.

## Highlights

• Proteomic analysis of FFPE specimens from four soft tissue sarcoma histological subtypes was performed by SWATH MS.
• Subtype-specific biological pathways and protein-protein interaction networks were identified.
• A panel of proteins associated with sarcoma patient survival outcome was defined.
• A subset of proteins identified by SWATH MS was validated by orthogonal immunohistochemical analysis.

## Graphical abstract

**Figure:**
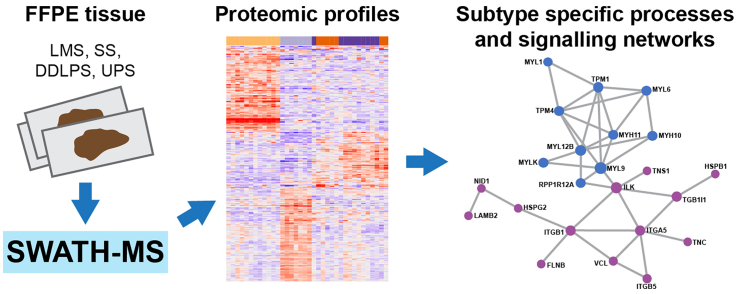

## Significance

Soft tissue sarcomas (STS) are rare tumours that account for 1% of adult cancers and comprise of >100 histological subtypes. While genomic and transcriptomic analyses of multiple subtypes have been reported, few comprehensive proteomic analyses by mass spectrometry have been undertaken. Herein we used sequential window acquisition of all theoretical fragment ion spectra mass spectrometry (SWATH-MS) to characterise a cohort of 36 formalin fixed paraffin embedded (FFPE) specimens from STS patients across four major histological subtypes. Proteomic profiling data defines underlying biological pathways enriched in each histological subtype including both well-established and new signalling networks of functional importance. A subset of proteins were subjected to orthogonal validation using immunohistochemistry. Further analysis of the proteomic dataset identifies a panel of 133 proteins associated with patient outcome in the STS cohort under study. We anticipate the future application of SWATH-MS to additional STS subtypes and larger cohorts has the potential to facilitate diagnosis, deliver new therapeutic targets and define prognostic signatures in these rare and difficult-to-treat diseases.

## 1 Introduction

STS are a group of rare and heterogeneous malignancies of mesenchymal origin, comprising more than 100 distinct diagnostic subtypes that are primarily defined by histological characteristics [1]. Multiple gene expression-based studies across different histological subtypes have led to a deeper understanding of the oncogenic processes driving STS development and progression as well as the identification of new prognostic signatures for these cancers [[2], [3], [4], [5]]. Whilst current diagnostic evaluation of STS is reliant on histological assessment by specialist sarcoma histopathologists, supplemented by specific molecular tests in selected subtypes, recent large-scale genomic and epigenetic analyses have demonstrated the feasibility of refining the classification system of STS based on intrinsic underlying biology [3,4]. Collectively, these studies highlight the promise of molecular profiling strategies in improving our knowledge of drivers of sarcoma pathogenesis, providing complementary information to aid in the molecular classification of these heterogeneous tumours, identify new therapeutic targets and develop clinically relevant prognostic biomarkers.

While the advent of next generation sequencing (NGS) has accelerated the use of genomic and epigenetic profiling in STS, proteomic analysis in this disease has been limited [6]. Comprehensive analysis of the tumour proteome is highly informative as proteins represent the largest class of druggable targets and directly reflect the functional state of biological pathways [7]. Proteomic analysis of STS has been performed by The Cancer Genome Atlas (TCGA) consortium in a cohort of 173 flash-frozen sarcoma specimens across six histological subtypes [3]. However, this study utilised the reverse phase protein array (RPPA) platform which is limited to the analysis of 192 proteins/phosphoproteins. Other studies that utilise mass spectrometry (MS) have largely been limited to 1–2 subtypes including recent proteomic analyses of gastrointestinal stromal tumours (GIST) and undifferentiated pleomorphic sarcomas (UPS) which identified distinct clinical and molecular subgroups [6,8,9]. To date few MS-based analysis has been conducted across multiple histological subtypes in STS.

In this study, we utilise sequential window acquisition of all theoretical fragment ion spectra (SWATH)-MS to undertake proteomic profiling of FFPE tissue specimens in a cohort of STS patients across four histological subtypes (*n* = 36). SWATH-MS is a next-generation proteomics method that offers high reproducibility in protein identification across multiple samples [10]. Due to the stochastic nature of precursor ion selection for fragmentation in conventional proteomic approaches such as data-dependent acquisition (DDA) mass spectrometry, there are routinely many missing values in large-scale proteomic studies, resulting in a reduction in the reproducibility of protein identification and quantification. In contrast, SWATH-MS selects and fragments all detected precursor ions in a sample generating a highly reproducible digital proteome map for each specimen in the cohort. Herein we characterise the intrinsic biological pathways associated with specific histotypes and explore the association of proteomic data with patient outcome. This study represents, to our knowledge, the most comprehensive analysis of the proteome in multiple STS subtypes to date and highlights the potential of proteomic profiles as predictors for patient outcome in these rare cancers.

## 2 Material and methods

### 2.1 Patients and tumour specimens

Use of archival FFPE tumour samples and linked anonymised patient data was approved by Institutional Review Board as part of the PROSPECTUS study, a Royal Marsden-sponsored non-interventional translational protocol (CCR 4371, REC 16/EE/0213). FFPE tissue from surgically resected primary tumours from four STS subtypes (LMS, SS, UPS and DDLPS) and accompanying annotation of baseline clinicopathological variables were identified and retrieved through retrospective review of departmental database and medical notes at a single specialist cancer centre. In line with standard management approaches, primary LMS, UPS and DDLPS tumours were naïve to any pre-operative therapy, while pre-operative exposure to chemotherapy and/or radiotherapy was varied in SS. The histological diagnosis was confirmed in all cases by experienced soft tissue pathologists (KT, CF). For each tumour, a single FFPE tissue block containing representative viable tumour was selected through review of haematoxylin and eosin (H&E)-stained sections. 20 μm sections were cut from each selected tumour block and, where indicated, macrodissected to enrich to >75% viable tumour content. In liposarcoma tumours that contained both well-differentiated and de-differentiated components, slide review and macrodissection ensured the dedifferentiated areas were sampled.

### 2.2 Protein extraction and sample preparation

20 μm tissue sections from each sample were deparaffinised by three washing steps in xylene, rehydrated by washes with decreasing ethanol gradient (100%, 96%, 70%) and then dried in a SpeedVac concentrator (Thermo Scientific). The lysis buffer (0.1 M Tris-HCl pH 8.8, 0.50% (*w*/*v*) sodium deoxycholate, 0.35% (w/v) sodium lauryl sulphate) was added at a ratio of 200ul/mg of dry tissue. The sample was homogenised using a LabGen700 blender (ColeParmer) with 3× 30s pulses and sonicated on ice for 10 min, then heated at 95 °C for 1 h to reverse formalin crosslinks. Lysis was carried out by shaking at 750 rpm at 80 °C for 2 h. The sample was then centrifuged for 15 min at 4 °C at 14,000 rpm and the supernatant collected. Protein concentration in the homogenate was measured by bicinchoninic acid (BCA) assay (Pierce) The extracted protein sample was digested using the Filter-Aided Sample Preparation (FASP) protocol as previously described in [11]. Briefly, each sample was placed into an Amicon-Ultra 4 (Merck) centrifugal filter unit and detergents were removed by several washes with 8 M urea. The concentrated sample was then transferred to Amicon-Ultra 0.5 (Merck) filters to be reduced with 10 mM dithiothreitol (DTT) and alkylated with 55 mM iodoacetamide (IAA). The sample was washed with 100 mM ammonium bicarbonate (ABC) and digested with trypsin overnight (Promega, trypsin to starting protein ratio 1:100 μg). Peptides were collected by two successive centrifugations with 100 mM ABC and desalted on C18 SepPak columns (Waters). The desalted peptide samples were then dried in a SpeedVac concentrator and stored at −80 °C.

### 2.3 SWATH-MS data acquisition and processing

Samples were resuspended in a buffer of 2% ACN/ 0.1% formic acid, spiked with iRT calibration mix (Biognosys AG) and analysed on an Agilent 1260 HPLC system (Agilent Technologies) coupled to a TripleTOF 5600+ mass spectrometer with NanoSource III (AB SCIEX). 1 μg of peptides for each sample was loaded onto a ZORBAX C18 (Agilent Technologies) trap column and separated on a 75 μm × 15 cm long analytical column with an integrated manually pulled tip packed with Reprosil Pur C18AQ beads (3 μm, 120 Å particles, Dr. Maisch). A linear gradient of 2–40% of Buffer B (98% ACN, 0.1% FA) in 120 min and a flow rate of 250 nl/min was used. Each sample was analysed in 2 technical replicates. Full profile MS scans were acquired in the mass range of *m/z* 340–1400 in positive ion mode. 8 data points per elution peak were set up for calculation of 60 precursor isolation windows with a fixed size of 13 Da across the mass range of *m*/*z* 380–1100 with 1 Da overlap. MS/MS scans were acquired in the mass range of *m/z* 100–1500. Maximum filling time for MS scans was 250 ms and for MS/MS scans 100 ms, resulting in a cycle time of 3.1 s. SWATH spectra were analysed using Spectronaut 11 (Biognosys AG) against a published human library [12]. FDR was restricted to 1% and only the top 6 peptides were used for quantification of a protein. Peak area of 2 to 6 fragment ions was used for peptide quantification, and the mean value of the peptides was used to quantify proteins. 2 peptides were set as minimum requirement for inclusion of a protein in the analysis.

### 2.4 Data processing and statistical methods

Data were log2 transformed, quantile normalised at sample level, followed by feature level (protein) centering across the samples to remove technical bias such as batch effect. Hierarchical clustering and Principle Component Analyses were used as visualisation tools to assess presence of batch effects. In brief, quantile normalisation was applied to transform the statistical distributions across samples to be the same, based on the assumption that the statistical distribution of each sample should be the same (or have the same distributional shape) within biological groups or conditions, but allowing that they may differ between groups. This procedure was performed using proBatch package in R [13]. Feature level (median centering) was performed in order to remove further technical bias. The proteomics data was visualised using 3D- t-Distributed Stochastic Neighbour Embedding. To assess similarity of protein expression profiles, pairwise Pearson correlations between all samples were visualised using the ‘ComplexHeatmap’ R package [14]. Dendrograms were created using complete linkage clustering and the Euclidean distances between the resulting correlations. Differential expression analysis was performed using Significant Analysis of Microarray upon a false discovery rate (FDR) less than 0.1% [15]. Gene Set Enrichment Analysis (GSEA) version 4.0.1 was used to identify gene sets from the Hallmark database (v7.0, ‘c5.bp.v7.0.symbols.gmt’) that were significantly enriched between each subtype and rest of the groups respectively. Protein-Protein Interaction (PPI) network analysis and visualisation was performed using NetworkAnalyst 3.0 pipeline against the STRING interactome database, with settings to confidence score > 900 and experimental evidence required [[16], [17], [18]]. The level 4 (log2 transformed with loading and batch corrected) RPPA dataset from the TCGA-SARC study was downloaded from The Cancer Proteome Atlas portal and clinical data downloaded from the TCGA Pan-cancer Clinical Data Resource (TCGA-CDR) within the NCI Genomic Data Commons [19,20]. The RPPA dataset was feature level (protein) median centred across samples and plotted along with the SWATH data using box-and-whisker plot. Univariable survival analysis of overall survival was estimated by Kaplan-Meier curve and Log-rank test for significance. Event was defined as death. Time was defined as surgery to death or last follow-up. Association between molecular groups and prognostic factors (grade, tumour size and sex) were tested by Fisher's exact test while association between molecular groups with age was tested by Kruskal-Wallis test. Univariable and multivariable Cox proportional hazards analyses were used to assess the prognostic significance of the different variables. Assessments of the proportional hazard were performed to check that the proportional hazard assumption is valid. Over-representation analysis was performed on the 133 identified proteins using PANTHER v14 [21].

### 2.5 Immunohistochemical staining and scoring

For validation of SWATH-MS results, the same cohort of specimens was stained for vinculin (ab219649, Abcam, 1:1000 dilution) and decorin (ab151988, Abcam, 1:500) in tissue microarray (TMA) format. To generate the TMAs, two to four 1 mm diameter cores were sampled from areas of viable tumour within FFPE donor blocks, re-embedded in an arrayed recipient paraffin block and sectioned at 4.0 μm thickness. Sections were deparaffinised in xylene and rehydrated using decreasing concentrations of ethanol in water (once in 100%, 96% and 80%). Antigen retrieval was performed in Tris-EDTA buffer (pH 6.0) for 8 min in a microwave oven and cooled for 45mins at room temperature. Sections were than washed once in Tris-buffered saline buffer (TBS), twice in Tris-buffered saline-Tween buffer (TBST) and incubated with blocking buffer for 90 min in a humidity chamber at room temperature. The blocking buffer is 3% (m/v) Bovine Serum Albumin (Sigma-Aldrich) in TBST. After blocking, sections were incubated with the primary antibody in a humidity chamber at 4 °C overnight.

The next day, slides were washed once in TBS and twice in TBST. Endogenous peroxidase activity was blocked with DAKO Peroxidase blocking solution (DAKO, Agilent Technologies) for 60 min at room temperature and sections were then washed once in TBS and twice in TBST. The sections were incubated with the goat anti-rabbit secondary antibody (7074S, Cell Signalling, 1:100) in the humidity chamber for 60 min at room temperature. The slides were washed again once in TBS and twice in TBST and then incubated with diaminobenzidine (DAKO, Agilent Technologies) for 25 s and 40s for vinculin and decorin staining, respectively. The sections were rinsed in water, counterstained with Modified Mayer's haematoxylin (Abcam) and dehydrated by taking them through washes in graded ethanol (once in 80%, 96% and 100%) and xylene. Finally, the slides were mounted in Pertex mounting medium (Pioneer) and scanned on Hamamatsu Nanozoomer slide scanner.

Scanned TMA cores were independently evaluated on a semi-quantitative basis by three investigators. Staining intensity was scored on the scale from 0 to 3 where 0: no staining; 1: weak staining; 2: moderate staining; 3: strong staining. Staining representation in the tumour area was scored on the scale from 0 to 3 where 0: <1% of tumour cells stained; 1: 1–10% of tumour cells stained; 2: 10–50% tumour cells stained; 3: >50% of tumour cells stained. Total score for each core was calculated as a sum of intensity and representation scores. For each case, median value of total scores was calculated across all cores scored by all three investigators and cases were further classified by a three tier system where “no or low staining”: total score median of 0–1; “weak staining”: total score median of 2–4; “strong staining”: total score median of 5–6. Borderline median scores (e.g. 1.5) were resolved by reviewing the IHC stained TMA cores and reaching a consensus classification between the three investigators.

### 2.6 Availability of data and materials

The MS proteomics data have been deposited to the ProteomeXchange Consortium via the PRIDE [22] partner repository with the dataset identifier PXD019719.

## 3 Results

### 3.1 Patient characteristics

The cohort is comprised of FFPE tumour material from 36 patients treated at The Royal Marsden Hospital. These specimens were obtained from surgical resections of primary tumours from four of the more common STS subtypes: leiomyosarcoma (LMS) (*n* = 12), synovial sarcoma (SS) (*n* = 7), UPS (*n* = 10) and dedifferentiated liposarcoma (DDLPS) (n = 7). Baseline clinico-pathological characteristics of these patients are summarised in Table 1.

**Table 1:** Clinico-pathological characteristics of STS cohort.

		Overall	LMS	SS	UPS	DDLPS
Number of cases		36	12	7	10	7
Age		62	67	49	76	61
		(28–84)	(35–75)	(43–77)	(28–84)	(37–78)
Gender	Female	25	12	4	5	4
	Male	11	0	3	5	3
Anatomical site	Intracavity	14	5	2	2	5
	Limbs	12	3	4	4	1
	Trunk/head	6	0	1	4	1
	Uterus	4	4	0	0	0
Disease stage	Localised	36	12	7	10	7
	Local recurrent	0	0	0	0	0
Histological grade	1	0	0	0	0	0
	2	16	6	4	4	2
	3	20	6	3	6	5
Pre-op treatment	No treatment	32	12	3	10	7
	Chemotherapy	1	0	1	0	0
	Chemo & Radiotherapy	3	0	3	0	0

### 3.2 Quantitative analysis of the STS proteome

**Fig. 1:**
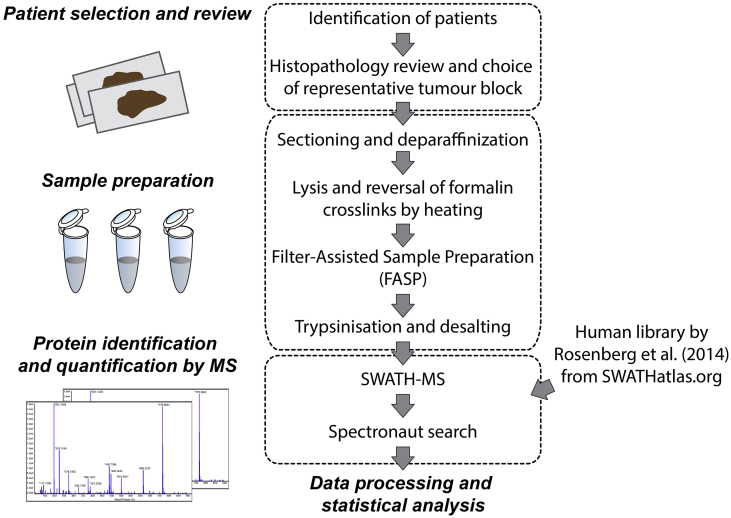
Schematic of the experimental workflow highlighting the key steps that were undertaken for sample selection and preparation as well as SWATH-MS data acquisition and analysis.

**Fig. 2:**
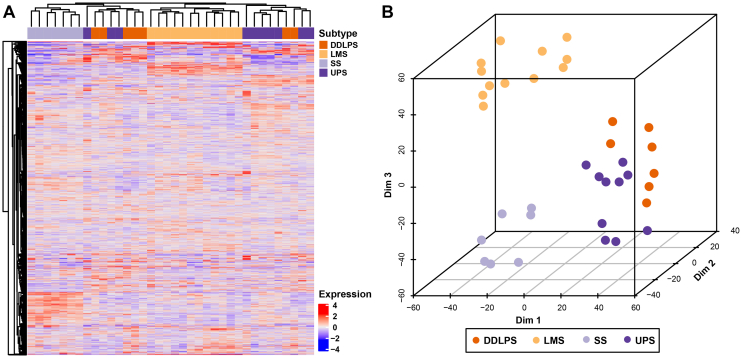
(A) Hierarchical clustering of 2951 proteins across 36 STS cases. The full list of proteins is listed in Table S1. (B) 3D-tSNE plot depicts four distinct groups of STS cases corresponding to the distinct histological subtypes. LMS is leiomyosarcoma, SS is synovial sarcoma, UPS is undifferentiated pleomorphic sarcoma and DDLPS is dedifferentiated liposarcoma.

STS specimens were subjected to protein extraction and SWATH-MS analysis in technical duplicates as outlined in Fig. 1. This analysis led to the identification and quantification of 2951 proteins across all samples (Fig. 2A and Table S1). Comparisons of quantitative data between technical duplicates in each sample resulted in a median Pearson's correlation coefficient of 0.994 (range 0.968–0.998), demonstrating exceptional reproducibility of the SWATH-MS methodology. Applying t-Distributed Stochastic Neighbour Embedding (t-SNE) on the full dataset, four major groups can be visualised on the 3D-tSNE plot (Fig. 2B), which correspond to the four distinct histological subtypes. These results demonstrate that SWATH-MS profiling can reveal intrinsic biology associated with distinct histological subtypes which are characterised by unique proteomic signatures.

### 3.3 Defining biological processes that are enriched in STS histological subtypes

**Fig. 3:**
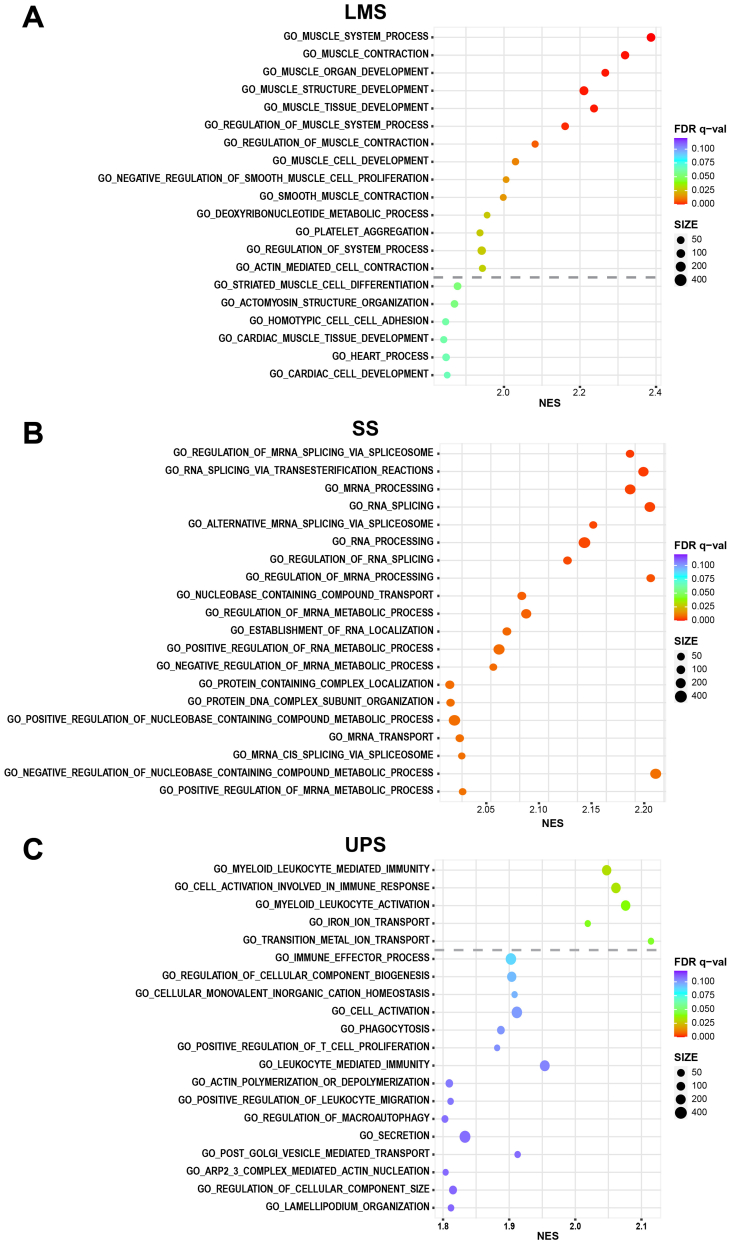
Plot of Gene Set Enrichment Analysis results showing the top ranked 20 positively enriched gene sets for (A) leiomyosarcoma (LMS), (B) synovial sarcoma (SS) and (C) undifferentiated pleomorphic sarcoma (UPS). The dashed line indicates a False Discovery Rate (FDR) = 0.05 threshold. The colour of the circles represents the FDR q-value while the size of the circle indicates the number of proteins within the dataset that is in each gene set. NES is the normalised enrichment score from the GSEA analysis.

To gain an understanding of the underlying gene set annotations in each histological subtype, we undertook gene set enrichment analysis (GSEA) of the full proteomic dataset [23]. The top 20 ranked enriched gene sets are shown in Fig. 3. Gene sets significantly enriched in LMS versus the rest of the cohort comprise of those involved in muscle development and contraction. This finding is consistent with the smooth muscle lineage of this histological subtype which has also been reported in published gene expression studies of LMS [2,24]. The utility of SWATH-MS data to rediscover known molecular processes highlights the validity of this approach for pathway discovery. In the case of SS, GSEA identifies RNA splicing and processing to be key gene sets significantly enriched in this histological subtype. This result is in line with a previous study which showed that the SS18-SSX fusion in SS binds to the ribonucleoprotein SYT-interacting protein/co-activator activator (SIP/CoAA) which is a key modulator of RNA splicing [25]. Meanwhile, gene sets significantly enriched in UPS finds a number of biological processes linked to leukocyte activation and metal ion transport. Notably, Phase II STS clinical trials of immune checkpoint inhibitors have reported evidence of clinical activity in UPS [26,27], underscoring the value of SWATH-MS in identifying biologically meaningful gene sets with potential clinical utility. No ontologies were found to be significantly enriched in DDLPS.

### 3.4 Identification of key protein complexes and signalling networks operating in STS histotypes

**Fig. 4:**
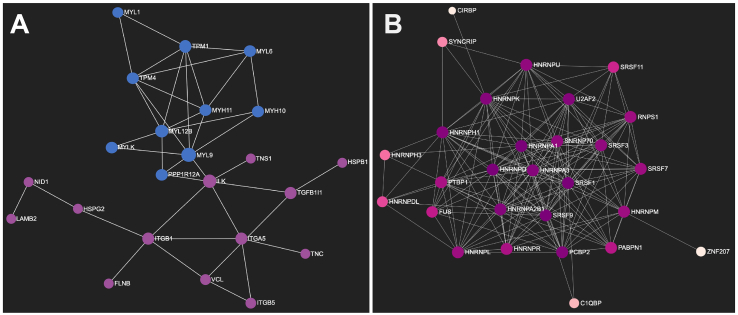
Network diagrams in Force Atlas layouts depicting protein-protein interaction maps for proteins which are significantly upregulated in (A) leiomyosarcoma and (B) synovial sarcoma. In (A), proteins in blue are components involved in the regulation of smooth muscle contraction while those in purple are components of the matrisome and adhesome. In (B), the majority of significantly upregulated proteins in synovial sarcoma are involved in RNA splicing regulation and the different colours indicate the number of interactions between identified proteins. (For interpretation of the references to colour in this figure legend, the reader is referred to the web version of this article.)

**Supplementary Figure S1:**
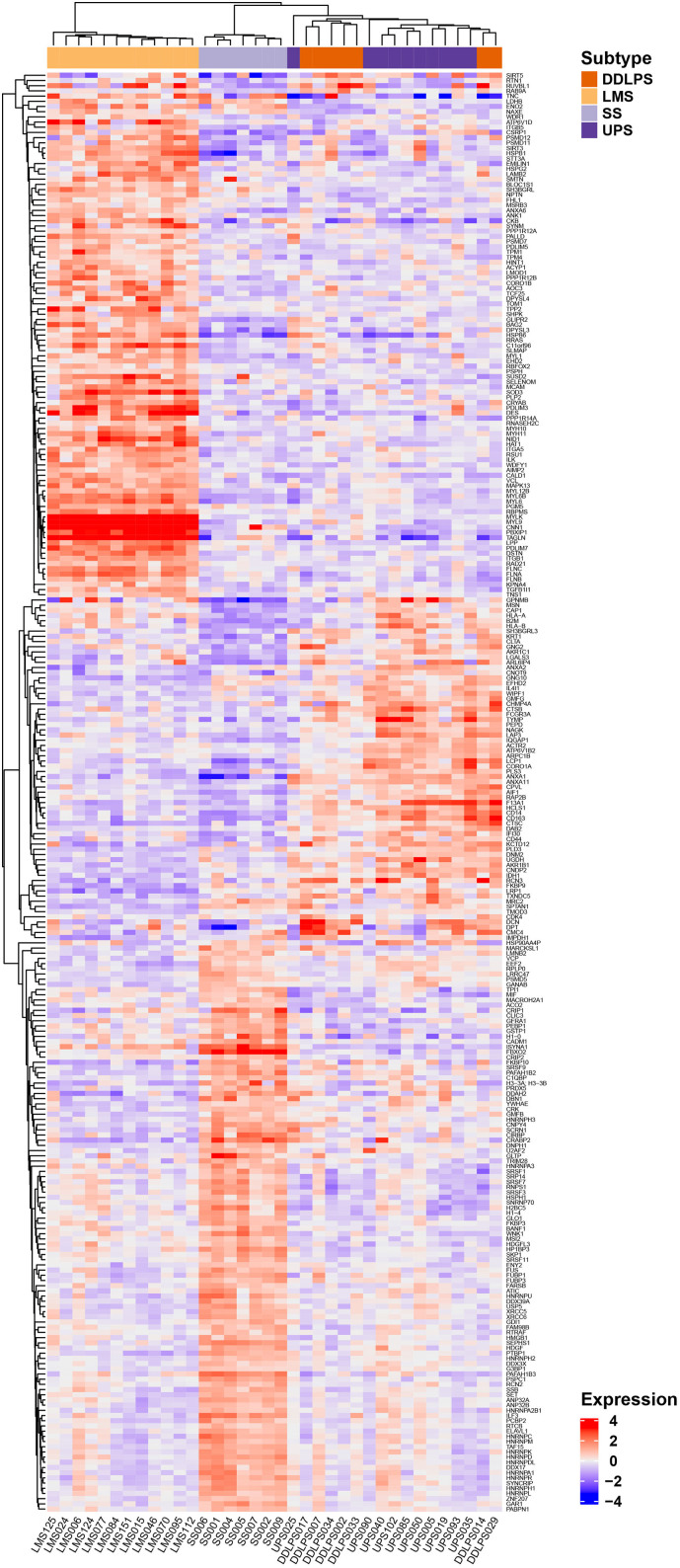
(A) Hierarchical clustering of 277 proteins that were found to be significantly differentially expressed across the four histotypes by multiclass Significance Analysis of Microarray (SAM) method (FDR <0.1%) across 36 STS cases. The full list of proteins is listed in Table S2. LMS is leiomyosarcoma, SS is synovial sarcoma, UPS is undifferentiated pleomorphic sarcoma and DDLPS is dedifferentiated liposarcoma.

**Supplementary Figure S2:**
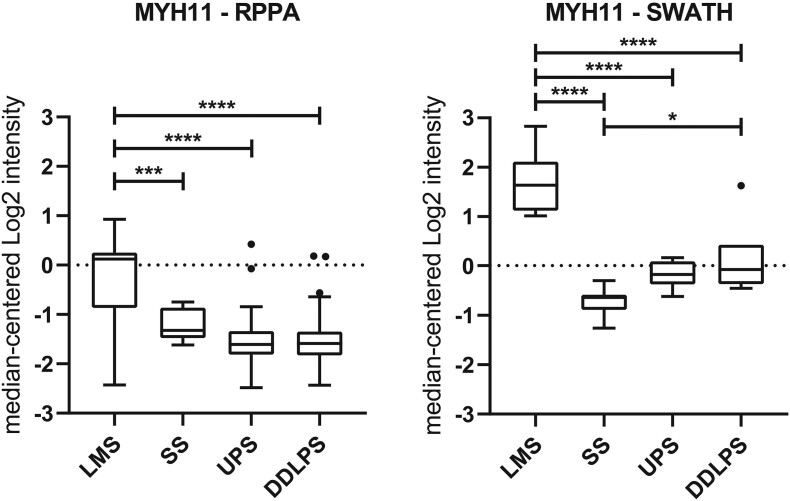
Boxplots comparing expression profile of MYH11 between reverse phase protein array (RPPA) dataset from the TCGA-SARC study and SWATH-MS dataset (SWATH) across 4 sarcoma subtypes. Boxplots shows 1st quartile, 3rd quartile and median value for each subtype, whiskers indicate interquartile range. Outliers are indicated by circle. Statistical significance is indicated by an asterisk where: * *p* < 0.05; ** *p* < 0.01; *** *p* < 0.001; **** *p* < 0.0001.

Using the multiclass Significance Analysis of Microarray (SAM) method, 277 proteins (FDR <0.1%) were found to be significantly differentially expressed across these four histotypes (Fig. S1, Table S2 and S3). Of these proteins, 95 were found to be significantly upregulated only in LMS, and 23 seed proteins were significantly mapped to directly interact with each other to form a zero-order network according to the STRING interactome database (Fig. 4A). These 23 seed proteins formed a subnetwork including the core machinery required for the regulation of smooth muscle contractile activity such as the myosin light chains (MYL1, MYL6, MYL9, MYL12B), myosin heavy chains (MYH10, MYH11), tropomyosin alpha chains (TPM1, TPM4), Myosin Light Chain Kinase (MYLK) and Protein Phosphatase 1 Regulatory Subunit 12A or Myosin Phosphatase Target Subunit 1 (PPP1R112A) (Fig. 4A). Evaluation of the protein expression levels of the myosin heavy chain MYH11 in the RPPA dataset from the independent TCGA sarcoma cohort (DDLPS *n* = 54, LMS *n* = 81, SS *n* = 6, UPS *n* = 49) confirms that this protein is significantly upregulated in LMS, providing independent validation of our SWATH-MS results (Fig. S2, Table S4) [3]. A second protein subnetwork comprising extracellular matrix components (matrisome) and their cognate adhesion receptors and downstream regulatory proteins (adhesome) was also found to be upregulated in LMS. This network includes the integrin receptors (ITGA5, ITGB1, ITGB5), intracellular adhesion signalling proteins (VCL, FLNB, ILK, TNS1) and extracellular matrisome components (HSPB1, HSPG1, NID1, LAMB2, TNC, TGFB1I1) [28,29] (Fig. 4A).

In SS, 103 proteins were found to be uniquely significantly upregulated; 28 of which were identified as seed proteins that significantly mapped to directly interact with each other to form a zero-order network. Twenty-four of these proteins are key components of mRNA splicing regulation. Cross-referencing these proteins to the SpliceosomeDB of spliceosome components [30] showed that the proteins enriched in SS comprise of the SR protein class (SRSF1, SRSF3, SRSF7, SRSF9, SRSF11) involved in constitutive and alternative pre-mRNA splicing, the heterogeneous ribonucleoprotein particle (hnRNPs) class and proteins involved in the formation of the spliceosome A Complex (HNRNPA1, U2AF2, SNRNP70) (Fig. 4B). Twenty-nine proteins were found to be upregulated only in UPS, and 3 seed proteins directly interact with each other, all components of the MHC class 1 complex (HLA-A, HLA-B and B2M). In DDLPS, 13 proteins were uniquely upregulated with no zero-order network identified. Notably, CDK4 is one of these 13 upregulated proteins which is in agreement with the molecular pathology of this disease where CDK4 is amplified in ~90% of DDLPS; and CDK4/6 inhibitors (palbociclib, ribociclib and abemaciclib) are currently being evaluated in the treatment of this histological subtype [[31], [32], [33]].

### 3.5 Identification of proteomic profiles that are associated with STS patient outcome

**Fig. 5:**
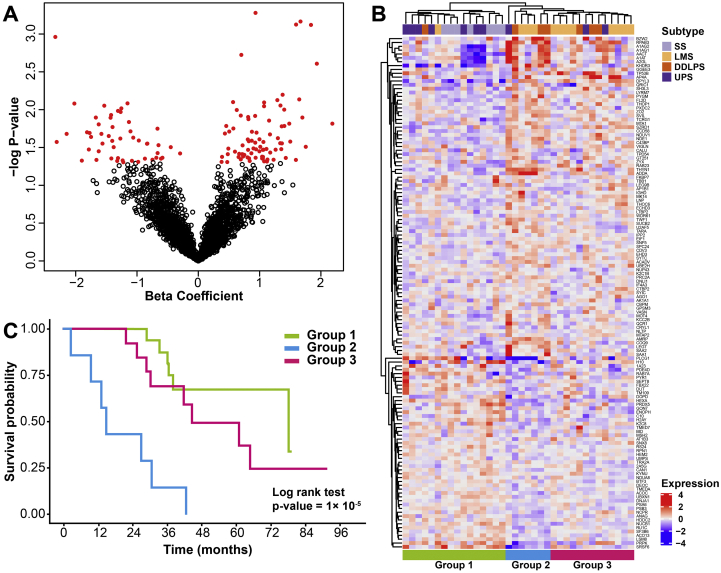
(A) Volcano plot of the beta coefficients from univariable Cox regression analysis for each of the 2951 proteins in the proteomic dataset and their associated –log *p*-value. Red circles indicate 133 proteins with *p* < 0.05. The full list of proteins is listed in Table S3. (B) Hierarchical clustering of 133 proteins across 36 STS cases identifies 3 subgroups of mixed histological subtypes. (C) The Kaplan Meier curves for overall survival (OS) of the 3 subgroups identified in (B). LMS is leiomyosarcoma, SS is synovial sarcoma, UPS is undifferentiated pleomorphic sarcoma and DDLPS is dedifferentiated liposarcoma. (For interpretation of the references to colour in this figure legend, the reader is referred to the web version of this article.)

In order to evaluate if there are proteins within our dataset that are associated with overall survival (OS) in our cohort of patients, univariable Cox regression analysis was performed for each of the 2951 proteins as exploratory analyses, and we selected a total of 133 proteins with *p* < 0.05 (Fig. 5A and Table S5). Subsequently, by hierarchical clustering of the 36 cases based on these 133 protein expression values, three subgroups of mixed histological subtypes were identified (Fig. 5B). These three groups were associated with significantly differential OS, with Group 2 comprising cases demonstrating the worst survival estimate (Fig. 5C, Log-rank *p* < 0.00001). There were no statistically significant associations between the molecular subgroups with prognostic factors grade, tumour size, sex and age (Table S6). However, there was a significant association between the molecular subgroup and sarcoma histological subtype (Table S6). Adjusting for other clinicopathological factors including age, tumour size, grade, sex and histological subtypes, the molecular subgroups remained an independent prognostic factor (Table S7) in the multivariable Cox regression analysis. After adjustment for other prognostic factors, patients in Group 2 were 72.6 times more likely to die (Hazard ratio 72.6; 95% Confidence Interval 3.71–1432; *p* = 0.005) compared to patients in Group 1 and 20.6 times more likely to die (Hazard ratio 20.6; 95% Confidence Interval 3.08–137; *p* = 0.002) compared to patients in Group 3. It should be noted that Group 2 is not a single cluster but is comprised of multiple smaller clusters. No statistically significant biological processes were identified to be enriched in the 133 proteins based on over-representation analysis [21].

### 3.6 Validation of vinculin and decorin protein expression by immunohistochemistry

**Fig. 6:**
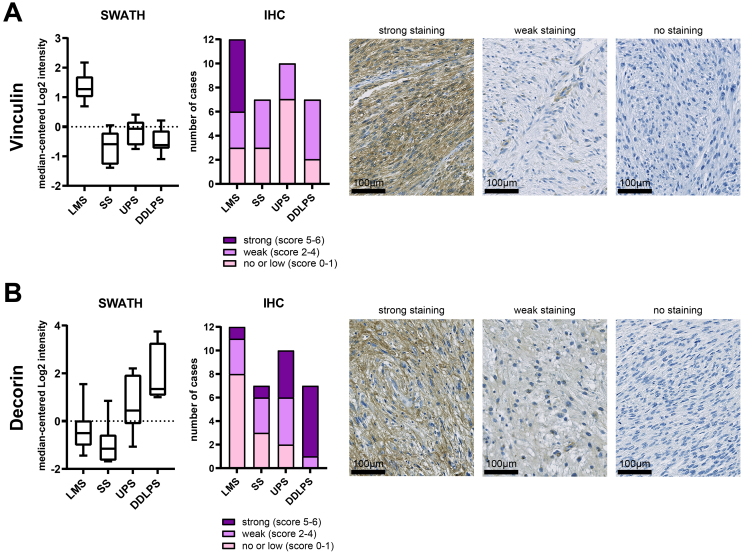
Comparison of expression profile of (A) vinculin and (B) decorin analysed by SWATH-MS (SWATH) with immunohistochemical (IHC) staining of TMA cores generated from the same tissue specimens in the cohort. Boxplots for SWATH-MS data shows 1st quartile, 3rd quartile and median value for each subtype, whiskers indicate interquartile range. Stacked bar charts indicate immunoreactivity of cases in each of the sarcoma subtypes. Photomicrographs of representative TMA cores with strong, weak and no staining for both proteins are shown.

To validate the SWATH-MS data using an orthogonal approach, we performed immunohistochemical (IHC) analysis of tumour cell expression levels of two proteins, vinculin (VCL) and decorin (DCN), in our cohort (Fig. 6). VCL is an intracellular adhesome signalling protein which was shown by SWATH-MS to be significantly upregulated in LMS compared to the other subtypes (Fig. 6A). Consistent with the SWATH-MS data, IHC analysis showed that half of all LMS cases (6/12) displayed strong tumour cell staining of this protein with the remaining cases either having weak (3/12) or no/low staining (3/12). This is in contrast to the other three subtypes where only weak or no/low staining was observed with no cases showing strong tumour cell expression of this protein. Decorin (DCN) is a proteoglycan that was found to be significantly upregulated in DDLPS compared to the other three histological subtypes (Fig. 6B). IHC analysis showed that 85% (6/7) of the DDLPS cases had strong protein expression levels of DCN with the remaining case having weak staining. In contrast, only a small proportion of cases in LMS (1/12, 8%), UPS (4/10, 40%) and SS (1/7, 14%) had strong tumour cell staining of this protein. Taken together, the IHC results of these two proteins are consistent with the SWATH-MS data which provides independent validation of the proteomic profiling dataset.

## 4 Discussion

In this study we have undertaken a comprehensive proteomic analysis of multiple STS subtypes and have demonstrated the utility of SWATH-MS in the following applications: 1. Defining unique proteomics signatures associated with distinct histological subtypes, 2. Identifying, within histological subtypes, biological processes and key protein networks and 3. Defining candidate proteins which are associated with predicting patient outcomes. Previous studies have shown that transcriptomic profiling can aid in the molecular classification of distinct sarcoma subtypes [34,35]. More recently, DNA methylation profiling has similarly revealed subtype-specific signatures that have utility in molecular classification, particularly in diagnostically challenging histological subtypes [[36], [37], [38]]. Our study shows for the first time that different histological subtypes harbour distinct proteomic signatures indicative of inherent subtype-specific biology driven by specific protein networks. We anticipate that expanded proteomic analysis incorporating larger STS cohorts with an increased number of histological subtypes will enable further refinement of this approach and the development of robust proteomic classifiers to aid in accurate molecular diagnosis of sarcomas.

In our study, we have identified a number of histotype-specific gene sets that are consistent with prior transcriptomic analyses. In LMS, ontology analysis of our proteomic data identifies biological processes associated with muscle development and contraction. Furthermore, analysis of protein-protein interactions identified a subnetwork comprising the core machinery involved in the regulation of smooth muscle contractile activity. This finding is in line with previous gene expression-based studies which have consistently demonstrated that smooth muscle functional gene ontologies are enriched in LMS cohorts from multiple independent transcriptomic datasets [4,24,39]. Interestingly, it has previously been shown that LMS can be classified into three distinct molecular subtypes based on transcriptomic data [24,39]. We anticipate that future studies integrating proteomics with transcriptomic analysis may shed light on the specific oncogenic pathways and candidate drug targets that are enriched in these LMS molecular subtypes which could be exploited for therapy. In UPS, our data finds that this subgroup is enriched in gene sets associated with leukocyte activation. This result is consistent with a very recent transcriptomic study which showed that UPS can been classified into two distinct molecular subgroups, one of which is characterised by genes involved in inflammatory response and immune cell signatures [9]. The enrichment of an immune cell proteomic signature in UPS, where immune checkpoint inhibitors have showed some clinical activity [26,27], suggests that future inclusion of proteomic profiling in cancer immunotherapy trials may lead to new predictive biomarkers that complement currently available approaches [40].

Several transcriptomic studies have led to the development of prognostic signatures in sarcoma. These include gene expression panels based on chromosome instability, hypoxia and stromal signatures [5,[41], [42], [43]]. Here we have performed an exploratory analysis which has for the first time identified a panel of proteins that is capable of stratifying a subgroup of patients (Group 2) with worse outcomes when compared with the rest of the cohort. Notably, Group 2 is composed of patients with mixed histological subtypes (3 DDLPS, 3 LMS and 1 UPS), and has a distinct expression profile of 133 proteins. Interestingly, Group 2 shared a subgroup of proteins that were also overexpressed within Group 3 (comprising mainly of DDLPS and LMS), while having low expression in a subset of proteins compared to both Group 1 and 3. These data suggest that these patients may share molecular characteristics that transcend histotypes. Over-representation analysis did not identify any enriched biological processes in the 133 proteins. It is important to note that this analysis was performed on a small patient cohort treated within a single institution and our findings should be considered hypothesis generating.

Together with a recently published SWATH-MS analysis in prostate cancer and diffuse large B-cell lymphoma [44], our study demonstrates the versatility of this label-free MS strategy in the analysis of FFPE specimens, a tissue preservation technique which has historically posed technical challenges for conventional proteomic workflows and analyses [45]. By utilising archival material from a tissue bank which is typically an abundant tissue resource, our study describing the proteomic profiling of STS from FFPE specimens opens new opportunities for gaining new insights into the biology and molecular drivers in rare cancers without the need for logistically challenging prospective collection of fresh tissue.

## 5 Conclusions

We have employed SWATH MS to profile FFPE tissue specimens across four sarcoma subtypes and have identified histotype-specific proteomic profiles that describe key biological pathways as well as discovered a panel of candidate proteins associated with patient outcome in STS. We anticipate the future application of this strategy to additional STS subtypes and clinical trial cohorts has the potential to deliver new therapeutic targets and define predictive and prognostic signatures in these rare and difficult-to-treat diseases.

The following are the supplementary data related to this article.

Supplementary Table S1 Supplementary Table S1

Supplementary Table S2 Supplementary Table S2

Supplementary Table S3 Supplementary Table S3

Supplementary Table S4 Supplementary Table S4

Supplementary Table S5 Supplementary Table S5

Supplementary Table S6 Supplementary Table S6

Supplementary Table S7 Supplementary Table S7

## Declaration of Competing Interest

The authors declare that they have no competing interests.
