# Supplementary material for: Proteomic profiling of soft tissue sarcomas with SWATH mass spectrometry
Source: J Proteomics. 2021 Jun 15;241:104236. doi: 10.1016/j.jprot.2021.104236 (PMC8135130; doi:10.1016/j.jprot.2021.104236)
Supplement: Supplementary Table S6 [file mmc8.docx]

**Table S6:** Association between molecular subgroups and clinicopathological factors.

| **Tested association** | **p-value** |
| --- | --- |
| Subgroup vs Grade | 0.19 |
| Subgroup vs Size | 0.78 |
| Subgroup vs Sex | 1 |
| Subgroup vs Subtype | **0.0004** |
| Subgroup vs Age | 0.35 |
