# Supplementary material for: Proteomic profiling of soft tissue sarcomas with SWATH mass spectrometry
Source: J Proteomics. 2021 Jun 15;241:104236. doi: 10.1016/j.jprot.2021.104236 (PMC8135130; doi:10.1016/j.jprot.2021.104236)
Supplement: Supplementary Table S7 [file mmc9.docx]

**Table S7:** Multivariable analysis of molecular subgroups and clinicopathological factors associated with outcome.

| Characteristic | HR^1^ | 95% CI^1^ | p-value |
| --- | --- | --- | --- |
| *Age* | 1.08 | 1.01 – 1.15 | 0.02 |
| *Subtype* |  | | |
| DDLPS | - | - |  |
| LMS | 0.21 | 0.02 – 2.17 | 0.2 |
| SS | 0.68 | 0.05 – 8.44 | 0.8 |
| UPS | 0.29 | 0.04 – 2.07 | 0.2 |
| *Size* |  | | |
| S | - | - |  |
| M | 0.2 | 0.04 – 1.08 | 0.061 |
| L | 0.25 | 0.03 – 2.26 | 0.2 |
| *Grade* |  | | |
| high | - | - |  |
| low | 0.62 | 0.17 – 2.19 | 0.5 |
| *Subgroup* |  | | |
| 1 | - | - |  |
| 2 | 72.6 | 3.71 – 1423 | **0.005** |
| 3 | 3.53 | 0.24 – 52.8 | 0.4 |
|  |  |  |  |
| 1 | 0.01 | 0.01 – 0.32 | **0.005** |
| 2 | - | - |  |
| 3 | 0.005 | 0 – 0.27 | **0.002** |
|  |  |  |  |
| 1 | 0.28 | 0.02 – 4.24 | 0.4 |
| 2 | 20.6 | 3.08 – 137 | **0.002** |
| 3 | - | - |  |
| *Sex* |  | | |
| F | - | - |  |
| M | 1.12 | 0.23 – 5.35 | 0.9 |

^1^ HR = Hazard Ratio, CI = Confidence Interval
